# The Protective Role of Coastal Marshes: A Systematic Review and Meta-analysis

**DOI:** 10.1371/journal.pone.0027374

**Published:** 2011-11-23

**Authors:** Christine C. Shepard, Caitlin M. Crain, Michael W. Beck

**Affiliations:** 1 Department of Ocean Sciences, University of California, Santa Cruz, California, United States of America; 2 Department of Ecology and Evolutionary Biology, University of California, Santa Cruz, California, United States of America; 3 The Nature Conservancy, Institute of Marine Sciences, University of California, Santa Cruz, United States of America; University of Western Australia, Australia

## Abstract

**Background:**

Salt marshes lie between many human communities and the coast and have been presumed to protect these communities from coastal hazards by providing important ecosystem services. However, previous characterizations of these ecosystem services have typically been based on a small number of historical studies, and the consistency and extent to which marshes provide these services has not been investigated. Here, we review the current evidence for the specific processes of wave attenuation, shoreline stabilization and floodwater attenuation to determine if and under what conditions salt marshes offer these coastal protection services.

**Methodology/Principal Findings:**

We conducted a thorough search and synthesis of the literature with reference to these processes. Seventy-five publications met our selection criteria, and we conducted meta-analyses for publications with sufficient data available for quantitative analysis. We found that combined across all studies (n = 7), salt marsh vegetation had a significant positive effect on wave attenuation as measured by reductions in wave height per unit distance across marsh vegetation. Salt marsh vegetation also had a significant positive effect on shoreline stabilization as measured by accretion, lateral erosion reduction, and marsh surface elevation change (n = 30). Salt marsh characteristics that were positively correlated to both wave attenuation and shoreline stabilization were vegetation density, biomass production, and marsh size. Although we could not find studies quantitatively evaluating floodwater attenuation within salt marshes, there are several studies noting the negative effects of wetland alteration on water quantity regulation within coastal areas.

**Conclusions/Significance:**

Our results show that salt marshes have value for coastal hazard mitigation and climate change adaptation. Because we do not yet fully understand the magnitude of this value, we propose that decision makers employ natural systems to maximize the benefits and ecosystem services provided by salt marshes and exercise caution when making decisions that erode these services.

## Introduction

Salt marshes provide humans many vital benefits known as ‘ecosystem services’ and one of the most important may be their role as buffers in protecting coastlines. Our coasts face a variety of natural hazards including storms, hurricanes, and tsunamis. These hazards are natural processes that have always affected the coastal zone, however the impacts and associated costs of these hazards to humans have increased as the amount and value of coastal infrastructure have grown and continue to grow. The effects of climate change will further amplify these impacts and costs. Sea level rise and ocean warming will increase the frequency and magnitude of many coastal hazards [1] while at the same time threatening coastal ecosystems such as salt marshes that humans are highly dependent upon.

Historically, coastal protection plans have relied on hardened infrastructure solutions such as sea walls, jetties and groins while ignoring or even destroying coastal marshes that could provide protective benefit. However, interest in natural or ecosystem-based coastal protection strongly increased after two recent natural disasters: the Indian Ocean tsunami and hurricane Katrina. Whereas the tsunami generated a great deal of inquiry into the protective role of mangroves [2], [3], [4], [5], [6], hurricane Katrina focused attention on the role of salt marshes in coastal protection [7], [8], [9]. After Katrina both the popular press and academic community quickly touted the importance of marshes for reducing storm surge waves and cited marsh loss as one culprit in the disaster. Many of the post-Katrina articles suggesting a link between salt marshes and surge reduction pointed to a 1963 US Army Corp of Engineers report that correlated storm surge elevations with over-marsh distance inland for seven storms crossing Louisiana between 1909 and 1957. While the frequently cited report does suggest that marshes can attenuate storm surge waves under some circumstances, nearly fifty years later we are only beginning to understand the role that marshes play in wave attenuation and more broadly in coastal protection.

In the aftermath of Katrina, the issue of whether or not marshes could attenuate extreme storm surge waves quickly became a contentious issue [10]. This focus on surge attenuation has overshadowed other, possibly more important, ecosystem services and potential protective benefits. For example, salt marsh vegetation has the potential to attenuate smaller more frequent waves and stabilize shorelines by promoting sediment deposition and reducing erosion. Additionally, salt marshes have the potential to mitigate flooding in coastal areas by reducing flood peaks and storing flood waters associated with coastal storms. Despite the potential significance of these processes for maintaining shorelines and attenuating coastal flooding, there has been only a limited consideration of the applicability of these services for mitigating current and future coastal hazards.

As the implications of climate change become clearer to coastal communities, there is mounting concern over the preparation and response to those growing hazards; this response is commonly known as climate change adaptation. As with coastal hazards mitigation, the first response in seeking adaptation solutions has been in gray or built infrastructure solutions. However, there is a nascent but growing interest in identifying where ecosystem-based approaches fit into these solutions and determining when and where they can help provide protection. Ecosystem-based or green solutions are an important component of calls for strengthened collaboration between the hazard mitigation and climate change adaptation research communities [11], especially with respect to incorporating the protective services of coastal wetlands [12], [13], [14]. Salt marshes, in particular, are a practical choice for inclusion in mitigation and adaptation approaches as marshes occupy much of the same low lying coastal areas that are especially vulnerable to sea level rise. Under certain circumstances, salt marshes may even be able to maintain the coastline relative to sea level rise by accreting sediment at a level comparable to or even higher than sea level rise providing a further reduction in vulnerability to hazards and climate change [15], [16].

In order to ascertain the utility of salt marsh ecosystem service provisioning for coastal planners and managers and to inform decision making related to ecosystem-based adaptation, we provide the most thorough synthesis of the protective benefits of salt marshes to date. We address three specific ecosystem services associated with coastal protection: *wave attenuation*, *shoreline stabilization*, and *floodwater attenuation*. For each service we performed an extensive search of the literature to identify primary research studies assessing the capacity for salt marshes to perform the service(s). We quantified service provision and recorded marsh vegetation characteristics and environmental factors that were associated with service provision. For services with sufficient studies (wave attenuation and shoreline stabilization), we conducted meta-analyses to assess the overall degree to which salt marshes perform each service, and where possible we did sub-analyses to examine how subgroups of studies performed differently. When meta-analysis was not possible, we quantified the frequencies of service provision across a range of salt marsh types and geographies to quantitatively summarize the evidence.

## Methods

### Searching

We searched the literature using the Biosis Previews, Web of Science, and Aquatic Sciences and Fisheries Abstracts databases (1900–2010, cutoff date May 15, 2010) to target articles related to the coastal protection services of wave attenuation, floodwater attenuation, and shoreline stabilization, defined below.

*Wave attenuation* is the reduction in wave energy or wave height that occurs when a wave passes through marsh vegetation. The energy of waves, tides, and currents is attenuated via frictional drag introduced by vegetation and by bottom friction in shallow water areas maintained by marshes [17], [18], [19]. Our search for articles explicitly evaluating wave attenuation within marshes was conducted using the search terms *marsh*, *wave*, *flow*, *attenuation* and *storm*.

*Shoreline stabilization* describes the processes by which salt marsh vegetation promotes sediment deposition, increases marsh elevations through below ground production, and stabilizes marsh sediments. The seaward salt marsh edge is linked to marsh elevation as a minimum elevation must be maintained to prevent marsh plant drowning and subsequent marsh edge loss. As a result, processes that maintain marsh elevation can also help maintain marsh shorelines and reduce erosion. Sediment deposition within marshes accounts for a large portion of elevation gains on the marsh surface along with small contributions from below ground processes such as root production [20], [21]. Subsidence and compaction can also affect the elevation of the marsh surface, particularly in rapidly subsiding marshes [22]. Belowground biomass, including roots and rhizomes, has been shown to reinforce the substrate and increase the shear strength of the soil potentially reducing erosion [23], [24]. To assess the capacity of marshes to provide shoreline stabilization, we surveyed the literature for articles explicitly evaluating accretion, marsh elevation changes, or erosion within marshes using the search terms *marsh*, *accretion*, *deposition*, *sedimentation*, and *erosion*.

*Floodwater attenuation* describes the capacity of salt marshes to reduce flood peaks or durations through storage and drainage of floodwaters. It is well known that marshes have a significant influence on the hydrological cycle both in terms of water quality and water quantity. However, the majority of this understanding lies in riparian or inland systems. In 1999, Bullock and Acreman [25] reviewed the literature to synthesize the evidence related to freshwater wetlands and the hydrological cycle. For 23 of the 28 identified studies evaluating freshwater wetlands and flooding, the authors found that floodplain wetlands reduced or delayed floods. While the floodwater attenuation capacity of wetlands along a river makes intuitive sense, the flood attenuation capacity of complex coastal marshes is likely not as straightforward. According to the United States Environmental Protection Agency (EPA), a one-acre wetland can on average store about three-acre feet of water, or one million gallons [26]. Although this value is a general value for a nondescript ‘wetland’, it reflects the likelihood that the storage capacity of coastal marshes may have the potential to reduce flood water heights and lessen flood related damages in the coastal zone. To identify studies evaluating the capacity of salt marshes to attenuate floodwaters, we used the search terms *marsh*, *flood*, *coastal flooding*, *water storage*, and *flood control*.

### Selection

We were specifically looking for studies that examined the given service in a controlled or paired experiment where the service was measured within and outside of the marsh vegetation. Where studies examined the degree to which vegetation promoted the service (either by manipulating vegetation type, density, productivity, etc.) the studies were collected to examine correlates of service provisioning. Correlates of service provisioning were categorized as marsh vegetation characteristics or hydrodynamic and physical environmental characteristics. For each of the three ecosystem services, we used two levels of screening to identify appropriate articles. For the first level of screening, abstracts were reviewed for the following exclusions: languages other than English, publication of abstract only, and publications not chiefly evaluating one or more of the three coastal protection services in a field or laboratory setting. Model studies were not included. Full text articles were obtained for all publications that passed the first level of screening and for publications in which a decision could not be based solely on the abstract. The second level of screening excluded full text publications without original data or analysis related to 1.) provision of services in vegetated versus unvegetated areas or 2.) variation in provision of services within vegetation. For each service, we recorded the number of records identified, number of studies included and excluded, and the reasons for exclusions according to the Preferred Reporting Items for Systematic Reviews and Meta-Analyses (PRISMA) Statement [27], see Figure S1.

### Data abstraction

We extracted qualitative and quantitative data from identified studies and divided them into studies that compared the service inside and outside of marsh vegetation versus those that examined the service only within vegetation. For the studies comparing vegetated and unvegetated treatments, we recorded whether each study found that marsh vegetation increased, decreased, or had no effect on the service and where possible recorded the statistical significance. When available, we also extracted mean responses for each service, sample sizes, and within system error estimates. Overall means for the vegetated and unvegetated treatments were either provided directly in the text or extracted from figures. In some cases, the presentation of raw data or means with no reported error measurements required us to calculate means and standard deviations. For example, sedimentation was sometimes measured at vegetated and unvegetated sites that we pooled to calculate means and error if a minimum of three sites was reported (n = 3) per treatment. In cases where data was reported over time, we used cumulative estimates where possible, or selected the last sampling date as the point of comparison. Additional information was collected on duration of study, weather conditions, vegetation type, response metric, and geographic location. Ideally vegetated and unvegetated areas were paired within a marsh system in generally equivalent elevations. However, in some cases (mostly for the wave attenuation studies), the vegetated and unvegetated sites were located within different elevation zones.

For wave attenuation, we designated percent wave height reduction per unit distance as the response variable. The typical method used to evaluate wave attenuation in the field is to measure incoming wave energy at wave recording stations along a shore normal transect that encompasses both vegetated and unvegetated areas. Laboratory based studies follow a similar approach by measuring wave energy or wave height passing through real or synthetic vegetation in a flume. We also extracted transect length and incident wave heights to evaluate the effects of these variables on attenuation.

In the case of shoreline stabilization, three response variables were accepted including accretion, surface elevation change, and lateral erosion. The terms accretion, sedimentation, and deposition are frequently used interchangeably and although they generally describe sediment being deposited on the surface, they are often measured and reported differently. Sedimentation and deposition are typically measured using sediment traps, petri dishes, and filter papers to record sediment deposition over time. Accretion is usually measured by laying down a marker horizon such as feldspar or clay and then measuring the vertical thickness of sediment deposited on top of the marker layer over time. Because sedimentation, accretion, and deposition are reflective of primarily surface processes, we grouped measurements of accretion, sedimentation, and deposition into the response measure ‘accretion’. We excluded historical studies using anthropogenic radionuclide-derived chronologies to reconstruct accretion rates. While constructing salt marsh sediment chronologies using anthropogenic radionuclides is an established technique, the resulting chronologies may not be reliable indicators of contemporary processes, particularly when evaluating erosion prone areas [28]. In addition, many reconstructed sediment chronologies estimate historic accretion within marshes but generally do not provide measurements under both control and experimental (vegetated and unvegetated) conditions.

Surface sediment level changes measured relative to a permanent or semi-permanent elevation benchmark were categorized as ‘marsh surface elevation’ measurements and were frequently measured using a Sedimentation Erosion Table. The table is used to measure changes in the sediment surface level attributable to both surface and subsurface processes [29]. Subsurface processes such as root production, shallow subsidence and compaction can contribute to elevation changes on the surface [20]. Our final response variable for shoreline stabilization, ‘lateral erosion’ was assessed by measuring changes in horizontal shoreline position and/or mass of eroded material. For each shoreline stabilization study, we extracted the response type, response units, and measurement procedure. We also recorded vegetation characteristics, soil types and hydrodynamic conditions for each study.

Because the floodwater attenuation search yielded so few relevant studies, quantitative analysis was not possible. For each identified publication, we extracted the spatial scale, location, and conclusions to qualitatively evaluate patterns in overall findings.

For wave attenuation and shoreline stabilization, each publication was further screened to identify factors, such as elevation or vegetation density, that are frequently correlated with service provision. For each publication, the factors that authors stated as being important drivers of variation in service provision were recorded. For each factor, we recorded whether or not the reported factor was shown to have a statistically significant effect and also the nature of the relationship (i.e. positive, negative, no effect).

### Quantitative data synthesis

Meta-analysis is a statistical technique for assessing the magnitude of a treatment effect by combining results from independent experiments. Effect size was calculated using weighted Hedges' *d*. For each service, we calculated the effect of salt marsh vegetation for each study (*d*) by calculating the difference between the means of the vegetated and unvegetated groups divided by the pooled standard deviation and weighted by a correction factor that adjusts for small sample size bias [30]. Overall effect of marsh vegetation was estimated by combining effect sizes across studies for each service. Studies were only included in the meta-analysis if they reported the statistical information necessary for calculating *d* (means, standard deviations, and sample sizes). We designated five studies as the minimum cutoff for performing meta-analysis. Thus, overall effect size was calculated only for wave attenuation and shoreline stabilization. For shoreline stabilization, a sufficient number of quantitative studies were available to examine the effect of vegetation on each response variable using meta-analysis. Salt marsh vegetation was considered to have a statistically significant effect on a given response variable if the 95% confidence interval (CI) did not overlap zero.

## Results

### Wave attenuation

**Figure 1:**
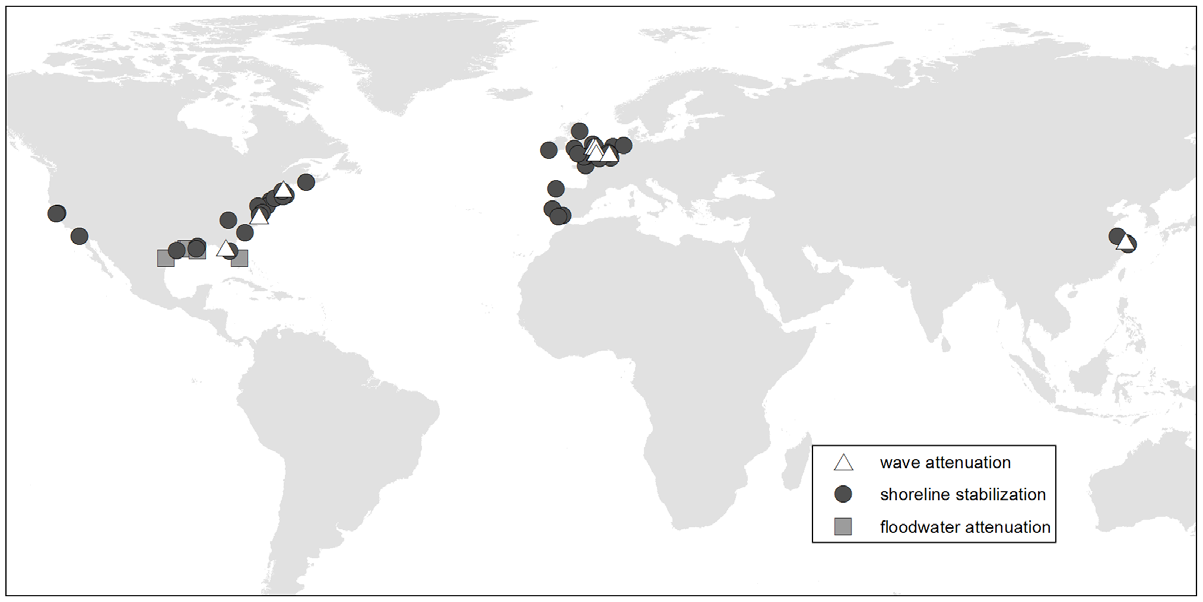
Global distribution of field studies evaluating coastal protection services provided by salt marshes.

The initial literature search identified 3285 publications for screening. Of these, 3187 abstracts were found to be largely unrelated to wave attenuation (likely a result of the wide-ranging search terms) and were rejected. Ten publications could not be retrieved prior to the retrieval cutoff. Of the remaining 88 articles, 74 did not meet our inclusion criteria which left 14 studies for analysis (Figure S2). Eleven studies were field based and three measured wave attenuation within a flume. Figure 1 shows the global distribution of field sites for the identified studies.

**Figure 2:**
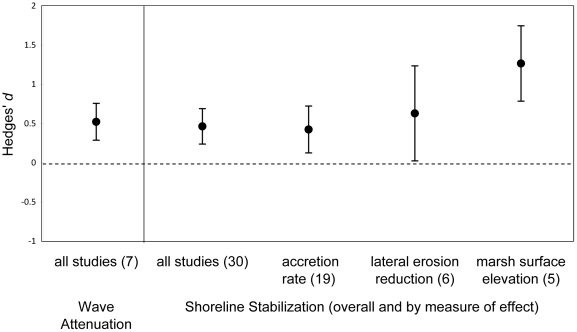
Average effect size of marsh vegetation (*d*) for meta-analyses on a.) wave attenuation and b.) shoreline stabilization as measured by increases in accretion/marsh surface elevation change or decreases in lateral erosion.

**Figure 3:**
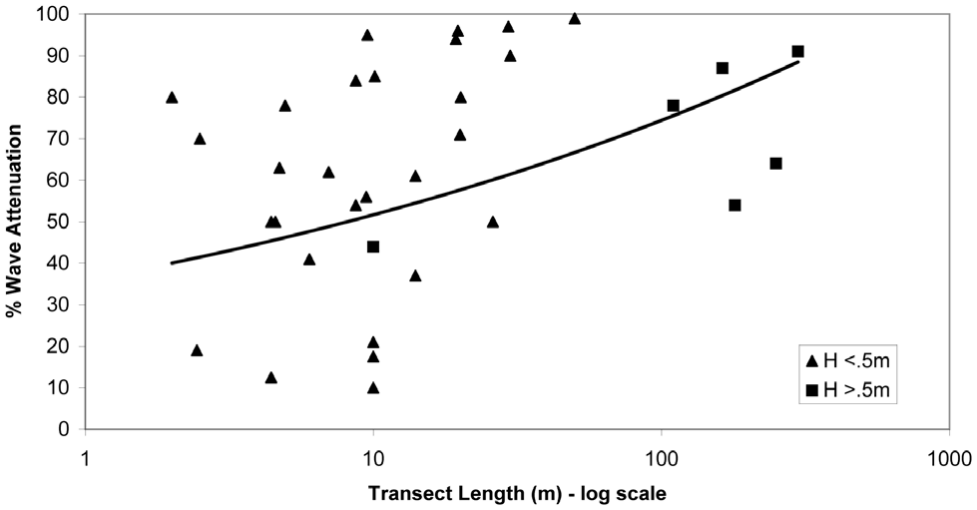
Reported wave attenuation rates through salt marsh vegetation versus marsh transect length. In most cases, the initial wave recorder was located at the marsh edge although some initial measurements were made just inside of the marsh edge. (H = wave height).

Ten studies examined wave attenuation rates per unit distance in both mud flats and adjacent salt marsh vegetation, while the remaining four studies provided wave attenuation estimates only within marsh vegetation. All ten studies comparing vegetated and unvegetated areas concluded that wave attenuation is greater across marsh vegetation than intertidal mudflat. Seven of these studies had sufficient detail to include in a meta-analysis (Table 1) that found a significant positive effect of vegetation on wave attenuation (d = 0.52±0.24, n = 7; Fig. 2). Wave attenuation rates generally increased with marsh transect length (Fig. 3). Attenuation rates for shorter transects (<10 m) were highly variable, but show that significant attenuation can occur even within the marsh edge.

**Table 1:** Characteristics and weighted effect sizes of studies included in the wave attenuation meta-analysis.

Species	Site Location	replicates within vegetation	replicates outside of vegetation	Weighted effect size (*d*)	Variance	Reference
*Spartina alt.*	Maine/New Hampshire	27	27	9.145	0.079	Morgan et al. 2009
*Schoenoplectus a.* *	Lake Christina, MN*	16	16	5.688	.0135	Allen et al. 2008
*Spartina alt.*	Chesapeake Bay, VA	4	3	1.060	0.628	Knutson et al. 1982
mixed	The Wash, UK	3	3	1.687	1.203	Cooper 2005
mixed	Norfolk Coast, UK	54	54	10.048	0.038	Möller et al. 1996, 1999
mixed	Tillingham, UK	19	19	3.973	0.108	Möller & Spencer 2002
mixed	Bridgewick, UK	23	23	4.708	0.089	Möller & Spencer 2002

(*d*) is found by calculating the difference between the means of the vegetated and unvegetated groups divided by the pooled standard deviation and weighted by a correction factor that adjusts for small sample size bias. Positive values indicate that marsh vegetation increases wave attenuation. Full citations are available in Figure S2.

* = Given the small number of studies evaluating wave attenuation, we included one freshwater study evaluating a species that can be found in coastal marshes. This species is also morphologically similar to many salt marsh species.

**Figure 4:**
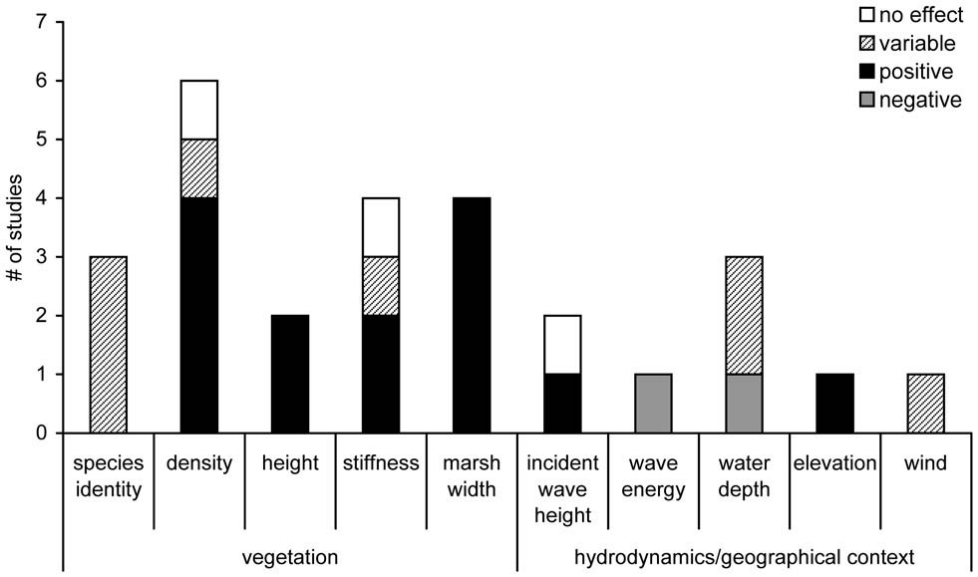
The factors most commonly quoted as being of importance to the wave attenuation capacity of salt marsh vegetation. The height of each bar indicates the number of author statements or conclusions regarding each factor and the bar fill indicates the effect of an increase in each factor on wave attenuation. For example, four studies showed that increasing marsh width increases wave attenuation indicating a positive relationship (black fill) between marsh width and wave attenuation.

Frequently identified factors that authors stated as being important determinants of wave attenuation within salt marshes were vegetation characteristics such as vegetation density, vegetation stiffness, and marsh width (Fig. 4). Marsh width and vegetation height showed a consistent positive effect on wave attenuation (Fig. 4). Author conclusions regarding hydrodynamic factors were reported less frequently however increasing wave energy and marsh elevations were identified as generally decreasing and increasing wave attenuation, respectively.

### Shoreline stabilization

The literature search identified 2330 citations for initial screening. 2225 publications were available for review and 2086 were rejected after reviewing the abstracts. Similar to wave attenuation, a large number of largely irrelevant studies were identified and rejected due to the breadth of our search terms. We obtained full text publications for the remaining 239 citations; only 57 publications met our criteria for inclusion (Figure S2). The majority of the experiments were conducted in the field (n = 53) with most studies taking place in North America, Europe, and China (Fig. 1).

**Figure 5:**
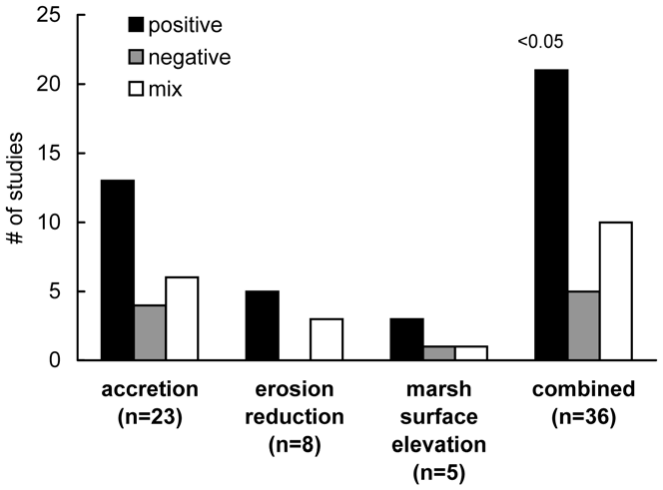
Reported outcomes from all shoreline stabilization publications comparing accretion, erosion reduction, or marsh surface elevation change in both vegetated and unvegetated salt marsh areas. The numbers of outcomes associated with each trend are plotted for each measure of shoreline stabilization and also all measures combined: gray bars represent a negative effect on the process, white bars represent no effect or mixed results and black bars represent a positive effect on the process. P value above the combined bars is significance value for chi square test. P values not reported for individual measures of effect due to insufficient frequency cell counts.

Thirty-three studies compared vegetated and unvegetated areas, yielding 36 independent comparisons of the effect of vegetation on one of the three measures of shoreline stabilization (Fig. 5). Accretion was the most frequently evaluated response (64% of studies), followed by erosion (22%) and elevation change (14%). Across all studies, a positive effect of marsh vegetation (increased accretion/surface elevation or reduced erosion) was reported in 58% of studies (Chi-square test, P<0.05, Fig. 5).

Of the 33 studies comparing vegetated and unvegetated areas, 18 studies and 38 independent measures of accretion, erosion, or surface elevation change had sufficient quantitative information for inclusion in a meta-analysis (Table 2). Across all studies, the overall effect of vegetation on shoreline stabilization was positive (d = 0.44±.22, n = 38). A positive and significant effect was also identified when we looked only at the most rigorous studies that controlled for tidal elevation between vegetated and unvegetated sites (d = 0.46±.23, n = 30; fig. 2). The effect of vegetation on each individual response variable was significantly positive (Fig. 2).

**Table 2:** Characteristics and weighted effect sizes of studies included in the shoreline stabilization meta-analysis.

Species	Site Location	Measure	replicates within vegetation	replicates outside of vegetation	Weighted effect size (*d*)	Variance	Reference
*Spartina foliosa*	Tijuana Estuary	AC	15	6	3.260	0.251	Ward et al. 2003
*Spartina foliosa*	Tijuana Estuary	AC	6	6	0.000	0.333	Ward et al. 2003
*Spartina alt.*	Nauset Marsh, MA	SEL	4	4	−1.393	0.548	Erwin et al. 2006
*Spartina alt.*	Little Beach, NJ	SEL	3	3	1.531	2.347	Erwin et al. 2006
*Spartina alt.*	Wachapreague, VA	SEL	3	3	−1.280	3.880	Erwin et al. 2006
*Spartina alt.*	Mockhorn, VA	SEL	2	2	−0.761	1.498	Erwin et al. 2006
*Spartina anglica*	Humber Estuary, UK	AC	5	5	1.916	0.444	Brown et al 1998
*Spartina anglica*	Humber Estuary, UK	AC	5	5	−3.190	0.764	Brown et al 1998
*Spartina anglica*	Humber Estuary, UK	AC	5	5	−0.996	0.410	Brown et al 1998
*Spartina anglica*	Humber Estuary, UK	AC	5	5	1.486	0.424	Brown et al 1998
*Spartina anglica*	Humber Estuary, UK	AC	5	5	3.031	0.609	Brown et al 1998
*Spartina anglica*	Humber Estuary, UK	AC	5	5	1.954	0.447	Brown et al 1998
mixed	Currituck Sound, NC	ER	4	4	1.207	0.534	Benner et al. 1982
mixed	Currituck Sound, NC	ER	4	4	1.764	0.589	Benner et al. 1982
mixed	Currituck Sound, NC	ER	4	4	0.805	0.514	Benner et al. 1982
mixed	Currituck Sound, NC	ER	4	4	−0.435	0.504	Benner et al. 1982
*Puccinellia m.*	Mt St. Michel Bay, France	SEL	8	9	2.171	6.256	Langlois et al. 2003
*Puccinellia m.*	Mt St. Michel Bay, France	SEL	8	9	4.706	0.302	Langlois et al. 2003
*Puccinellia m.*	Mt St. Michel Bay, France	SEL	8	9	5.182	0.807	Langlois et al. 2003
*Spartina alt.*	Wallops Island, VA	SEL	30	14	6.183	0.110	Reidenbaugh et al. 1983
*Spartina alt.*	S.F. Bay, CA	AC	10	10	5.797	0.264	Neira et al. 2006
*Spartina alt.*	Great Sipp. Marsh, MA	AC	2	8	1.910	0.751	Jordan & Valiela 1983
*Spartina foliosa*	Tijuana Estuary	AC	22	23	−7.200	0.094	Wallace et al. 2005
*Spartina alt.*	Wave flume	ER	4	4	−0.854	0.516	Feagin et al. 2009
*Spartina alt.*	Galveston Island, TX	SEL	4	4	0.118	0.500	Feagin et al. 2009
*Juncus gerardi*	Tay Estuary, UK	AC	5	5	2.948	0.578	McManus & Alizai
*Spartina alt.*	Long Island, NY	AC	2	2	0.797	2.389	Richard 1978
*Spartina m.*	Tagus Estuary, Portugal	AC	5	5	−0.450	0.402	Salguiero & Cacador 2007
*Spartina m.*	Tagus Estuary, Portugal	AC	5	5	1.327	0.419	Salguiero & Cacador 2007
*Spartina m.*	Tagus Estuary, Portugal	AC	5	5	−2.766	0.533	Salguiero & Cacador 2007
*Spartina spp.*	Southampton Water UK	AC	2	2	1.520	6.641	Quaresma et al. 2007
*Spartina alt.*	Dongtai, China	AC	2	2	2.443	2.247	Chung et al. 2004
*Spartina alt.*	Dongtai, China	AC	4	4	0.734	0.512	Chung et al. 2004
*Spartina m.*	Ria Formosa, Portugal	AC	3	2	−0.958	1.010	Neumeier & Ciavola 2004
*Spartina m.*	Ria Formosa, Portugal	AC	6	3	−1.893	0.587	Neumeier & Ciavola 2004
mixed	Tagus Estuary, Portugal	AC	4	4	0.099	0.500	Silva et al. 2009
mixed	Castlemaine Harbor, Ire.	AC	5	5	2.638	0.512	Duffy & Devoy 1999
*Phragmites au.*	Wave flume	ER	6	6	3.686	1.006	Coops et al. 1996

(*d*) is found by calculating the difference between the means of the vegetated and unvegetated groups divided by the pooled standard deviation and weighted by a correction factor that adjusts for small sample size bias. Positive values of *d* indicate that marsh vegetation increases shoreline stabilization. Measures of shoreline stabilization include AC = Accretion, SEL = Marsh surface elevation change, ER = Lateral Erosion. The sign of *d* was reversed for studies that reported lateral erosion. Full citations are available in Figure S2.

**Figure 6:**
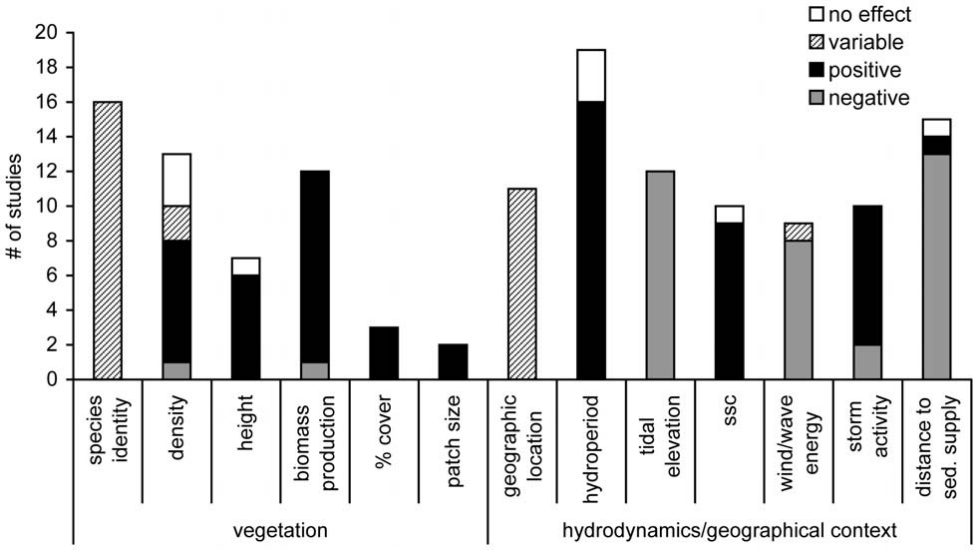
The factors most commonly quoted as being of importance to the shoreline stabilization capacity of salt marshes. An effect is positive if an increase in the factor results in an increase in shoreline stabilization as measured by increases in accretion or marsh surface height or a reduction in lateral erosion. The effect of an increase in each factor on shoreline stabilization is indicated by the bar fill.

Factors most frequently identified as being correlated with shoreline stabilization included vegetation characteristics such as species identity, vegetation density, vegetation height, and biomass production (Fig. 6). Characteristics of importance unrelated to marsh vegetation included hydroperiod (length of tidal inundation) and distance to a sediment supply such as a river or creek.

### Floodwater attenuation

We identified 2664 citations from the literature search and after reviewing abstracts, 2530 studies were rejected because they did not evaluate floodwater attenuation within marshes. We were unable to retrieve 13 publications prior to the retrieval cutoff which left 121 full text articles for review. Of the full text articles, none quantified floodwater storage or flood peak attenuation in a controlled or paired experiment within and outside of salt marsh vegetation. We did identify four studies evaluating the effects of marsh alteration on flooding at scales ranging from individual marsh areas to coastal watersheds (Fig. 1, Table 3).

**Table 3:** Studies providing support for the floodwater attenuation capacity of coastal marshes.

Study	Geographical Context	Findings
Bolduc and Afton 2004	Rockefeller State Wildlife Refuge, LA30,700 ha	Structural marsh management (alteration of hydrology) reduces drainage as compared to unimpounded marshes
Meeder 1987	Rainey Refuge, LA	Natural marsh areas drained quickly compared to areas adjacent to water control structures
Swenson and Turner 1987	Coastal marsh areas, LA40 ha	Partially impounded marsh flooded more than control, also reduced above and below ground water exchange
Brody et al. 2007	Coastal watersheds of Texas and Florida (fourth order watersheds)	Wetland alteration in coastal watersheds exacerbated flooding events

The four studies suggest that natural or unaltered coastal marshes drain water more quickly and effectively. Increased drainage in marsh areas (either to coastal water bodies or into the ground) may help store and drain waters away from adjacent developed areas. Full citations are available in Figure S2.

## Discussion

The meta-analyses and qualitative evaluations of the literature indicate that salt marsh vegetation has a significant positive effect on wave attenuation and shoreline stabilization and these results provide support for comprehensive approaches that incorporate natural features and processes into hazard mitigation and climate change adaptation. While previous individual studies have shown that marsh vegetation attenuates wave energy, the results of our meta-analysis show this to be the case across a range of geographic and hydrodynamic settings. Observation intervals for the wave attenuation studies ranged from a single wave to an extended time series of waves with wave amplitudes ranging from millimeters to just under one meter. It is important to understand how wave attenuation varies with changes in scale because salt marshes are exposed to multiple wave magnitudes and frequencies of exposure. The majority of studies evaluated small to moderate incoming waves; however these are the types of waves most frequently impacting salt marshes [31]. Positive overall effects were also found for shoreline stabilization with the magnitude of this effect being greatest for marsh surface elevation change. Marsh vegetation likely had the strongest effect on surface elevation gains due to its influence on vegetation capture, deposition of sediment above the surface, and belowground processes such as root growth [20], [21], [32].

In addition, correlates of service provision frequently identified by authors suggest important marsh and hydrodynamic characteristics related to coastal protection (Figs. 4 and 6). Vegetation characteristics such as high density, high biomass production, and large marsh size were identified as being important drivers for positively affecting both wave attenuation and shoreline stabilization. This overlap in significant drivers suggests that large marshes that contain dense and productive vegetation will attenuate wave energy and stabilize shorelines more effectively than deteriorating or severely altered marshes. Because these vegetation characteristics can exhibit seasonal changes related to plant growth, there is likely temporal variation in service provision [33]. Wave attenuation can also vary spatially across the marsh vegetation (Fig. 3). Attenuation rates within the first ten meters are variable but frequently exceed 50% highlighting the importance of marsh edge or fringing marshes for attenuation. Further, beyond transect lengths of 10 meters, wave attenuation increases non-linearly suggesting non-linearity in the provision of wave attenuation [34]. However, attenuation rates for the longer transects are likely conservative estimates as these values do not reveal where the majority of the attenuation takes place along the transect.

Hydrodynamic and physical environmental characteristics had inconsistent correlations with wave attenuation but were less frequently evaluated than for shoreline stabilization where hydroperiod, elevation, wave energy, and sediment availability were frequently identified as having consistent positive or negative effects. Suspended sediment concentrations and proximity to sediment supplies, both reflective of sediment availability, are particularly important because sediment availability is often reduced by human modifications aimed at increasing coastal protection (e.g. sea walls and levees) [35].

The effects of storms appear to be more complex. For example, increasing wind and wave energy (typical characteristics of storms) were frequently correlated with lower wave attenuation rates and reduced shoreline stabilization. In contrast, storm activity was often identified as a likely contributor to shoreline stabilization. This non-intuitive finding is likely due to different time scales of measurement as wave attenuation rates are measured immediately while elevation or erosion changes are measured over longer time periods. For wave attenuation rates measured during extreme wave or wind events, water levels may have increased to a point beyond which significant attenuation of wave energy by vegetation is likely. Shoreline stabilization processes measured during or immediately after a storm would also likely indicate a decrease in service provision (i.e. increased erosion) within vegetation. However, once the storm has moved through the area, suspended sediment often settles back onto the marsh surface. This re-deposited sediment can cause a significant amount of accretion or elevation gain following storms (see McKee and Cherry 2009 for summary) and in some cases this deposition stimulates belowground productivity [36].

Over the past 25 years, many models have been developed to better understand the relationships between hydrodynamics, elevation, and vegetation in salt marshes [37]. These range in scale from zero dimensional point based models to landscape models and many of these published models considering the effects of marsh vegetation are in agreement with our results (for example see [38], [39], [40]). Model based studies can also be helpful for understanding interactive effects that are difficult to examine in a field setting, for instance feedbacks between factors such as vegetation characteristics and coastal geomorphology. For example, Kirwan and Murray (2008) developed an ecogeomorphic model that considered a vegetation-related feedback function in which plant productivity affects sediment deposition which can affect elevation, which impacts vegetation productivity [41]. The continued development and improvement of these types of interactive models will allow us to better understand when and where salt marshes can be incorporated into coastal protection planning.

There is also evidence that in addition to mitigating high frequency, low magnitude coastal hazard events, marsh processes such as wave attenuation, sediment deposition and elevation building can also contribute to the long term maintenance of the coastline. This is especially relevant for areas with significant projected increases in sea level. Sea level rise varies spatially; this is often described as relative sea level rise (RSLR). Marsh areas experiencing high levels of subsidence, such as in the northern Gulf of Mexico, experience higher levels of RSLR [22], [42]. Although the effects of SLR on salt marshes are dependent on local factors and beyond the scope of this paper, in general a salt marsh can only persist if the surface elevation increases at a rate greater than or equal to RSLR. Cahoon et al. (2006) assessed the relationship between accretion, surface elevation change, and sea level rise across a sample of 78 coastal marshes in North America, Europe, and Australia. Their results showed that average accretion and elevation rates were greater than corresponding rates of RSLR demonstrating that many salt marshes are accreting sediment at a rate necessary to ‘keep up’ with RSLR [43]. However, it is difficult to extrapolate the response of coastal marshes to RSLR over time using short term (less than 5 year) records [20] and there is uncertainty regarding how SLR will impact the delivery of marsh ecosystem services [44], [45], [46].

Our literature search revealed critical research gaps related to storm surge and coastal flooding. Most of the identified wave attenuation studies evaluated small to moderate waves (Hs<.5 m) and there was a total lack of field studies quantitatively evaluating large waves and storm surge. This gap in our understanding is of critical importance because storm surge and associated flooding cause the majority of hurricane related damage and fatalities [47]. To date, the only field-based data for storm surge attenuation are observational attenuation rates derived from water level gauges placed within coastal marshes. Observations from one of the few studies conducted in this area suggested that every 14.5 km of marsh provides a 1 meter reduction in storm surge [48]. Similarly, a 1994 U.S. Geological Survey report [49] documented storm surge elevations decreasing across 37 meters of marsh and water at a rate of 1 meter per 20 km when Hurricane Andrew made its second landfall in Louisiana in 1992. These estimates were not included in our review because the values were generated with no consideration of topography, distance from the storm, or marsh properties. Wamsley et al (2009) attempted to control for some of these confounding factors when they calculated and then hindcasted attenuation rates for Hurricane Rita ranging from a 1 m reduction per 25 km to a 1 m reduction per 4 km. Although inland storm surge propagation is a very complex process dependent on many factors other than marsh length alone, Wamsley et al.'s results and other recent model-based studies suggest that healthy, intact coastal marshes can attenuate surge [50], [51], [52]. Though recent advances in modeling technology have allowed a more quantitative evaluation of the large scale effects of coastal marshes on storm surge, there is a substantial need for large-scale, field based studies evaluating attenuation of large waves (>1 m) and storm surge. Indeed, two of the model-based surge attenuation studies [51], [52] noted ongoing and recently completed field-based assessments of surge attenuation in coastal marshes.

Remarkably, what happens to storm surge water once it is pushed onshore has also not been adequately studied. As we have shown, there are a surprisingly limited number of studies that address the floodwater attenuation capacity of salt marshes and more are needed. Nonetheless, the papers we identified show a fairly clear pattern regarding the effects of marsh alteration on water quantity regulation. These studies provide evidence that natural marsh areas drain more efficiently compared to altered marsh areas and that wetland alteration increases flooding events on a regional scale. Thus it is very likely that salt marshes are providing some level of floodwater attenuation by absorbing water and moving water in a sheet flow towards the coast [53]. Increasing coastal development and wetland alterations will likely bring about more serious flooding due to further reductions in floodwater storage capacity and increased storm water runoff in developed areas from increases in impervious surface cover [54]. There is a need for more studies both during and after storm events to characterize the flood response of complex and often heavily modified coastal marshes. Others have noted this need and are in agreement with the importance of this ecosystem service for coastal protection despite the paucity of studies quantitatively evaluating this service [48], [51]. In fact, a recent study of the linkages between the oceans and human health noted a critical need for epidemiological research to address the public health consequences of coastal flooding and the anticipated amplification of this human health hazard due to climate change [55].

Although field-based studies demonstrate the effects of salt marsh vegetation on processes related to coastal protection, the extent to which each process translates into coastal protection is likely site dependent and related to interactive factors such as coastal geomorphology, marsh health and extent, and hydrologic regime. The protective value of coastal marshes may be best evaluated using innovative correlative techniques such as that employed by [56] to show the effects of wetland alteration on flooding. The authors evaluated the relationship between the number of granted wetland alteration permits and coastal watershed flooding over a 12 year period using a multivariate regression analysis. Their results showed that wetland alteration exacerbates flooding events in coastal watersheds even with the inclusion of control variables to account for socioeconomic, demographic, and environmental differences that might also influence the level of flooding. Regression based techniques have also been used to evaluate the effects of coastal wetlands on hurricane damage and fatalities. Costanza et al. [57] estimated the economic value of coastal wetlands in the United States for coastal protection and concluded that coastal wetlands currently provide 23.2 billion dollars per year in storm protection services. Similarly, Pérez-Maqueo et al. [58] found that area covered by semi-altered ecosystems (a mosaic of natural and human-altered ecosystems) and GDP negatively affect mortality rates associated with hurricanes. While these studies did not directly evaluate the mechanism by which wetlands attenuate storm waves or reduce damage to human communities, their results do provide insight into the economic and societal benefits of the protection services of coastal marshes.

This review and analysis strengthens the view that management of salt marshes should be integrated into coastal zone hazard mitigation and climate change adaptation policies. Salt marsh conservation and restoration decisions can and should be framed in the larger context of sustaining our coastlines and vice versa. One version of this is already taking place in parts of the United Kingdom and the Netherlands where managed realignment is being advocated to meet biodiversity and hazard mitigation objectives. Managed realignment is the practice of moving the coastal defense line (for example, a sea wall) inland to allow an area of previously reclaimed land to be re-inundated and develop intertidal habitat such as salt marsh [59], [60]. At least 24 managed realignment projects have been undertaken in the UK, with seven explicitly created to provide a combination of flood risk management and habitat creation benefits [60]. While managed realignment shows promise in the creation of new salt marsh habitat for coastal protection, persistence of existing coastal marshes will be determined by both anticipated climate change and the many past and future human impacts to marshes [35], [61], [62]. More than half of U.S. salt marshes (and their associated ecosystem services) have been lost due to direct and indirect human impacts. Human modifications to the coastal zone have resulted in decreased sediment supply to marshes, altered hydrological functioning, and increased subsidence all of which contribute to marsh loss and decrease coastal protection services. How we chose to respond to coastal hazards and sea level rise has further significant implications for sustaining our coastal livelihoods and ecosystems. It is clear that coastal management decisions should consider the dynamics of natural coastal systems previous to human modification and be cautious about any actions that erode the natural benefits and ecosystem services provided by salt marshes.

## Supporting Information

Figure S1 **PRISMA literature search flow diagrams.**
(DOC)

Figure S2 **publications included in review and meta-analysis.**
(DOC)
