# Supplementary material for: The Protective Role of Coastal Marshes: A Systematic Review and Meta-analysis
Source: PLoS One. 2011 Nov 23;6(11):e27374. doi: 10.1371/journal.pone.0027374 (PMC3223169; doi:10.1371/journal.pone.0027374)
Supplement: Figure S1 — PRISMA literature search flow diagrams. (DOC) [file pone.0027374.s001.doc]

Appendix S1. PRISMA Literature Search Flow Diagrams

**Wave Attenuation**

**Screening**

**Included**

**Eligibility**

**Identification**

Records identified through database searching
(n = 4089)

Additional records identified through other sources
(n = 2)

Records after duplicates removed
(n = 3285)

Records screened
(n = 3285)

Records excluded or unavailable
(n = 3197)

Full-text articles assessed for eligibility
(n = 88)

Full-text articles excluded, with reasons
(n = 74)

Studies included in qualitative synthesis
(n = 14)

Studies included in quantitative synthesis (meta-analysis)
(n = 7)

**Shoreline Stabilization**

**Screening**

**Included**

**Eligibility**

**Identification**

Records identified through database searching
(n = 3514)

Additional records identified through other sources
(n = 0 )

Records after duplicates removed
(n = 2330)

Records screened
(n =2330)

Records excluded or unavailable
(n = 2091)

Full-text articles assessed for eligibility
(n = 239)

Full-text articles excluded, with reasons
(n = 182)

Studies included in qualitative synthesis
(n = 57)

Studies included in quantitative synthesis (meta-analysis)
(n = 18 )

**Floodwater Attenuation**

**Screening**

**Included**

**Eligibility**

**Identification**

Records identified through database searching
(n = 2732)

Additional records identified through other sources
(n = 0)

Records after duplicates removed
(n = 2664)

Records screened
(n =2664)

Records excluded or unavailable
(n = 2543)

Full-text articles assessed for eligibility
(n = 121)

Full-text articles excluded, with reasons
(n = 117)

Studies included in qualitative synthesis
(n = 4)

Studies included in quantitative synthesis (meta-analysis)
(n = 0 )
