# Supplementary material for: The Protective Role of Coastal Marshes: A Systematic Review and Meta-analysis
Source: PLoS One. 2011 Nov 23;6(11):e27374. doi: 10.1371/journal.pone.0027374 (PMC3223169; doi:10.1371/journal.pone.0027374)
Supplement: Figure S2 — publications included in review and meta-analysis. (DOC) [file pone.0027374.s002.doc]

Appendix S2. Publications Included in Review and Meta-Analysis

**Wave Attenuation**

Allen JH, Nuechterlein GL, Buitron D (2008) Bulrush Mediation Effects on Wave Action: Implications for Over-water Nesting Birds. Waterbirds 31: 411-416.

Augustin LN, Irish JL, Lynett P (2009) Laboratory and numerical studies of wave damping by emergent and near-emergent wetland vegetation. Coastal Engineering 56: 332-340.

Bouma TJ, De Vries MB, Low E, Peralta G, Tanczos C, et al. (2005) Trade-offs related to ecosystem engineering: A case study on stiffness of emerging macrophytes. Ecology 86: 2187-2199.

Cooper NJ (2005) Wave Dissipation Across Intertidal Surfaces in the Wash Tidal Inlet, Eastern England. Journal of Coastal Research 21: 28-40.

Coops H, Geilen N, Verheij HJ, Boeters R, van der Velde G (1996) Interactions between waves, bank erosion and emergent vegetation: an experimental study in a wave tank. Aquatic Botany 53: 187-198.

Knutson PL, Brochu RA, Seelig WN, Inskeep MR (1982) Wave damping in Spartina alterniflora marshes. Wetlands 2: 87-104.

Möller I, Spencer T, French JR (1996) Wind wave attenuation over saltmarsh surfaces: Preliminary results from Norfolk, England. Journal of Coastal Research 12: 1009-1016.

Möller I, Spencer T (2002) Wave dissipation over macro-tidal saltmarshes: Effects of marsh edge typology and vegetation change. Journal of Coastal Research SI36: 506-521.

Möller I (2006) Quantifying saltmarsh vegetation and its effect on wave height dissipation: Results from a UK East coast saltmarsh. Estuarine Coastal and Shelf Science 69: 337-351.

Möller I, Spencer T, French JR, Leggett DJ, Dixon M (1999) Wave transformation over salt marshes: A field and numerical modelling study from north Norfolk, England. Estuarine Coastal and Shelf Science 49: 411-426.

Morgan PA, Burdick DM, Short FT (2009) The Functions and Values of Fringing Salt Marshes in Northern New England, USA. Estuaries and Coasts 32: 483-495.

Wayne (1976) The Effect of Sea and Marsh Grass on Wave Energy. Coasta Res Notes 4: 6-8.

Yang SL (1998) The role of Scirpus marsh in attenuation of hydrodynamics and retention of fine sediment in the Yangtze Estuary. Estuarine Coastal and Shelf Science 47: 227-233.

Yang SL, Li H, Ysebaert T, Bouma TJ, Zhang WX, et al. (2008) Spatial and temporal variations in sediment grain size in tidal wetlands, Yangtze Delta: On the role of physical and biotic controls. Estuarine Coastal and Shelf Science 77: 657-671.

**Shoreline Stabilization**

Benner CS, Knutson PL, Brochu RA, Hurme AK (1982) Vegetative erosion control in an oligohaline environment Currituck Sound, North Carolina. Wetlands 2: 105-117.

Bouma TJ, Friedrichs M, van Wesenbeeck BK, Temmerman S, Graf G, et al. (2009) Density-dependent linkage of scale-dependent feedbacks: a flume study on the intertidal macrophyte Spartina anglica. Oikos 118: 260-268.

Brown SL, Warman EA, McGrorty S, Yates M, Pakeman RJ, et al. (1998) Sediment Fluxes in Intertidal Biotopes: BIOTA II. Marine Pollution Bulletin 37: 173-181.

Castillo JM, Leira-Doce P, Rubio-Casal AE, Figueroa E (2008) Spatial and temporal variations in aboveground and belowground biomass of Spartina maritima (small cordgrass) in created and natural marshes. Estuarine Coastal and Shelf Science 78: 819-826.

Castillo JM, Luque CJ, Castellanos EM, Figueroa ME (2000) Causes and consequences of salt-marsh erosion in an Atlantic estuary in SW Spain. Journal of Coastal Conservation 6: 89-96.

Chung CH, Zhuo RZ, Xu GW (2004) Creation of Spartina plantations for reclaiming Dongtai, China, tidal flats and offshore sands. Ecological Engineering 23: 135-150.

Coops H, Geilen N, Verheij HJ, Boeters R, van der Velde G (1996) Interactions between waves, bank erosion and emergent vegetation: an experimental study in a wave tank. Aquatic Botany 53: 187-198.

Culberson SD, Foin TC, Collins JN (2004) The role of sedimentation in estuarine marsh development within the San Francisco Estuary, California, USA. Journal of Coastal Research 20: 970-979.

Davidson-Arnott RGD, van Proosdij D, Ollerhead J, Schostak L (2002) Hydrodynamics and sedimentation in salt marshes: examples from a macrotidal marsh, Bay of Fundy. Geomorphology 48: 209-231.

Duffy MJ, Devoy RJN (1999) Contemporary process controls on the evolution of sedimentary coasts under low to high energy regimes: western Ireland. Geologie En Mijnbouw 77: 333-349.

Erwin R, Cahoon DR, Prosser DJ, Sanders GM, Hensel P (2006) Surface Elevation Dynamics in Vegetated Spartina Marshes Versus Unvegetated Tidal Ponds Along the Mid-Atlantic Coast, Usa, with Implications to Waterbirds. Estuaries and Coasts 29: 96-106.

Esselink P, Dijkema KS, Reents S, Hageman G (1998) Vertical accretion and profile changes in abandoned man-made tidal marshes in the Dollard estuary, the Netherlands. Journal of Coastal Research 14: 570-582.

Feagin RA, Lozada-Bernard SM, Ravens TM, Moller I, Yeager KM, et al. (2009) Does vegetation prevent wave erosion of salt marsh edges? Proceedings of the National Academy of Sciences of the United States of America 106: 10109-10113.

French JR, Spencer T (1993) Dynamics of sedimentation in a tide-dominated backbarrier salt marsh, Norfolk, UK. Marine Geology 110: 315-331.

Gleason ML, Elmer DA, Pien NC, Fisher JS (1979) Effects of stem density upon sediment retention by salt marsh cord grass, Spartina alterniflora Loisel. Estuaries 2: 271-273.

Harrison EZ, Bloom AL (1977) SEDIMENTATION RATES ON TIDAL SALT MARSHES IN CONNECTICUT USA. Journal of Sedimentary Petrology 47: 1484-1490.

Houwing EJ (2000) Morphodynamic development of intertidal mudflats: consequences for the extension of the pioneer zone. Continental Shelf Research 20: 1735-1748.

Jordan TE, Valiela I (1983) SEDIMENTATION AND RESUSPENSION IN A NEW-ENGLAND SALT-MARSH. Hydrobiologia 98: 179-184.

Langley J, McKee KL, Cahoon DR, Cherry JA, Megonigal J (2009) Elevated CO sub(2) stimulates marsh elevation gain, counterbalancing sea-level rise. Proceedings of the National Academy of Sciences, USA 106: 6182-6186.

Langlois E, Bonis A, Bouzille JB (2003) Sediment and plant dynamics in saltmarshes pioneer zone: Puccinellia maritima as a key species? Estuarine Coastal and Shelf Science 56: 239-249.

Marion C, Anthony EJ, Trentesaux A (2009) Short-term ( approximately equal to 2 yrs) estuarine mudflat and saltmarsh sedimentation: High-resolution data from ultrasonic altimetery, rod surface-elevation table, and filter traps. Estuarine, Coastal and Shelf Science 83: 475-484.

McManus J, Alizai SAK (1987) VARIATIONS IN MARSH SURFACE LEVELS IN THE UPPER TAY ESTUARY SCOTLAND UK. Proceedings of the Royal Society of Edinburgh Section B (Biological Sciences) 92: 345-358.

Morris JT, Sundareshwar PV, Nietch CT, Kjerfve B, Cahoon DR (2002) Responses of coastal wetlands to rising sea level. Ecology 83: 2869-2877.

Neira C, Grosholz ED, Levin LA, Blake R (2006) Mechanisms generating modification of benthos following tidal flat invasion by a Spartina hybrid. Ecological Applications 16: 1391-1404.

Neumeier U, Giavola P (2004) Flow resistance and associated sedimentary processes in a Spartina maritima salt-marsh. Journal of Coastal Research 20: 435-447.

Nyman JA, Crozier CR, Delaune RD (1995) ROLES AND PATTERNS OF HURRICANE SEDIMENTATION IN AN ESTUARINE MARSH LANDSCAPE. Estuarine Coastal and Shelf Science 40: 665-679.

Oenema O, Delaune RD (1988) ACCRETION RATES IN SALT MARSHES IN THE EASTERN SCHELDT SOUTHWEST NETHERLANDS. Estuarine Coastal and Shelf Science 26: 379-394.

Pasternack GB, Brush GS (2002) Biogeomorphic controls on sedimentation and substrate on a vegetated tidal freshwater delta in upper Chesapeake Bay. Geomorphology 43: 293-311.

Proosdij DV, Ollerhead J, Davidson-Arnott RGD (2000) Controls on suspended sediment deposition over single tidal cycles in a macrotidal saltmarsh, Bay of Fundy, Canada. Geological Society, London, Special Publications 175: 43-57.

Quaresma V, Bastos AC, Amos CL (2007) Sedimentary processes over an intertidal flat: A field investigation at Hythe flats, Southampton Water (UK). Marine Geology 241: 117-136.

Ranwell DS (1964) Spartina salt marshes in southern England. II. Rate and seasonal patteFn of sediment accretion. J Ecol 52: 79-94.

Reidenbaugh TG, Varricchio M, Strieter RP, Mendoza S, Banta WC (1983) SHORT-TERM ACCRETIONAL AND EROSIONAL PATTERNS IN A VIRGINIA USA SALT MARSH. Gulf Research Reports 7: 211-216.

Rejmanek M, Sasser CE, Peterson GW (1988) HURRICANE-INDUCED SEDIMENT DEPOSITION IN A GULF COAST MARSH. Estuarine Coastal and Shelf Science 27: 217-222.

Richard GA (1978) SEASONAL AND ENVIRONMENTAL VARIATIONS IN SEDIMENT ACCRETION IN A LONG-ISLAND SALT-MARSH. Estuaries 1: 29-35.

Richards PJ (1934) The salt marshes of the Dovey Estuary. 4. The rates of vertical accretion, horizontal extension and scarp erosion. Ann Botany 48: 225-259.

Rooth JE, Stevenson JC (2000) Sediment deposition patterns in Phragmites australis communities: Implications for coastal areas threatened by rising sea-level. Wetlands Ecology and Management 8: 173-183.

Rosen PS (1980) Erosion susceptibility of the Virginia Chesapeake Bay shoreline. Mar Geol 59.

Rosso PH, Ustin SL, Hastings A (2006) Use of lidar to study changes associated with Spartina invasion in San Francisco Bay marshes. Remote Sensing of Environment 100: 295-306.

Salgueiro N, Cacador I. Short-term sedimentation in Tagus estuary, Portugal: the influence of salt marsh plants; 2007. pp. 185-193.

Sanchez JM, SanLeon DG, Izco J (2001) Primary colonisation of mudflat estuaries by Spartina maritima (Curtis) Fernald in Northwest Spain: vegetation structure and sediment accretion. Aquatic Botany 69: 15-25.

Schmitt C, Weston N, Hopkinson C (1998) Preliminary evaluation of sedimentation rates and species distribution in Plum Island Estuary, Massachusetts. Biological Bulletin, Marine Biological Laboratory, Woods Hole 195: 232-233.

Silva H, Dias J, Cacador I (2009) Is the salt marsh vegetation a determining factor in the sedimentation processes? Hydrobiologia 621: 33-47.

Temmerman S, Govers G, Wartel S, Meire P (2003) Spatial and temporal factors controlling short-term sedimentation in a salt and freshwater tidal marsh, Scheldt estuary, Belgium, SW Netherlands. Earth Surface Processes and Landforms 28: 739-755.

van Proosdij D, Davidson-Arnott RGD, Ollerhead J (2006) Controls on spatial patterns of sediment deposition across a macro-tidal salt marsh surface over single tidal cycles. Estuarine Coastal and Shelf Science 69: 64-86.

Wallace KJ, Callaway JC, Zedler JB (2005) Evolution of tidal creek networks in a high sedimentation environment: A 5-year experiment at Tijuana Estuary, California. Estuaries 28: 795-811.

Wang A, Gao S, Jia J (2006) Impact of the cord-grass Spartina alterniflora on sedimentary and morphological evolution of tidal salt marshes on the Jiangsu coast, China. Acta Oceanologica Sinica/Haiyang Xuebao 25: 32-42.

Ward KM, Callaway JC, Zedler JB (2003) Episodic colonization of an intertidal mudflat by native cordgrass (Spartina foliosa) at Tijuana Estuary. Estuaries 26: 116-130.

Widdows J, Brinsley M (2002) Impact of biotic and abiotic processes on sediment dynamics and the consequences to the structure and functioning of the intertidal zone. Journal of Sea Research 48: 143-156.

Widdows J, Pope ND, Brinsley MD (2008) Effect of Spartina anglica stems on near-bed hydrodynamics, sediment erodability and morphological changes on an intertidal mudflat. Marine Ecology-Progress Series 362: 45-57.

Wood N, Hine AC (2007) Spatial trends in marsh sediment deposition within a microtidal creek system, Waccasassa Bay, Florida. Coastal Research: 823-833.

Yang S-l (1999) Tidal wetland sedimentation in the Yangtze Delta. Journal of Coastal Research 15: 1091-1099.

Yang SL (1998) The role of Scirpus marsh in attenuation of hydrodynamics and retention of fine sediment in the Yangtze Estuary. Estuarine Coastal and Shelf Science 47: 227-233.

Yang SL (1999) A study of coastal morphodynamics on the muddy islands in the Changjiang River estuary. Journal of Coastal Research 15: 32-44.

Yang SL (1999) Sedimentation on a growing intertidal island in the Yangtze River mouth. Estuarine Coastal and Shelf Science 49: 401-410.

Yang SL, Ding PX, Chen SL (2001) Changes in progradation rate of the tidal flats at the mouth of the Changjiang (Yangtze) River, China. Geomorphology 38: 167-180.

Yang SL, Eisma D, Ding PX (2000) Sedimentary processes on an estuarine marsh island within the turbidity maximum zone of the Yangtze River mouth. Geo-Marine Letters 20: 87-92.

Yang SL, Friedrichs CT, Shi Z, Ding PX, Zhu J, et al. (2003) Morphological response of tidal marshes, flats and channels of the outer Yangtze River mouth to a major storm. Estuaries 26: 1416-1425.

**Floodwater Attenuation**

Bolduc F, Afton AD (2004) Hydrologic aspects of marsh ponds during winter on the Gulf Coast Chenier Plain, USA: effects of structural marsh management. Marine Ecology-Progress Series 266: 35-42.

Brody SD, Highfield WE, Ryu HC, Spanel-Weber L (2007) Examining the relationship between wetland alteration and watershed flooding in Texas and Florida. Natural Hazards 40: 413-428.

Meeder J (1987) Variable effects of hurricanes on the coast and adjacent marshes: A problem for marsh managers N V Brodtmann (ed), Proceedings of the Fourth Water Quality and Wetlands Management Conference Tulane University, New Orleans: 337–374.

Swenson EM, Turner RE (1987) Spoil Banks – Effects on a coastal marsh water-level regime. Estuarine Coastal and Shelf Science 24: 599-609.
